# Isolated tracheobronchial inflammation with airway obstruction: a diagnostic challenge

**DOI:** 10.1016/j.ero.2026.02.011

**Published:** 2026-03-12

**Authors:** Panagiota Alexopoulou, Konstantinos Kotsifas, Aikaterini Kaziani, Dimitrios Kalkanis, Anastasios Karamanakos

**Affiliations:** 1 Department of Rheumatology, Evangelismos General Hospital, Athens, Greece; 2 Pulmonary Medicine Department, Evangelismos General Hospital, Athens, Greece; 3 Department of Internal Medicine, Evangelismos General Hospital, Athens, Greece; 4 PET/CT Department, IATROPOLIS Group of Medical Companies, Athens, Greece

## CASE

A 56-year-old previously healthy male gymnast presented with a 2-month dry cough and a 1-month intermittent fever. He had voice hoarseness from a vocal cord polyp diagnosed 20 years ago.

At a previous hospital, a chest computed tomography (CT) scan showed sparse basal fibrosis, and laboratory tests indicated high inflammatory markers, with microbiological cultures and polymerase chain reaction for respiratory pathogens yielding negative results. Bronchioalveolar lavage revealed neutrophilic infiltration. He was empirically treated with broad-spectrum antibiotics due to persistent fever; he initially responded but relapsed after SARS-CoV-2 infection, treated with remdesivir and dexamethasone, although with mild symptoms, with transient fever relief.

After discharge, the fever reoccurred. A repeat chest CT showed diffuse thickening of the tracheal and bronchial walls. Fluorine-18 fluorodeoxyglucose positron emission tomography–CT (¹⁸F-FDG PET-CT) demonstrated uptake along the trachea and main bronchi, sparing the posterior tracheal circumference, with mild hypermetabolic mediastinal lymph nodes (Fig, A-C). Bronchoscopy revealed diffuse nodular thickening, oedema of the tracheal and bronchial mucosa, an enlarged main carina, and an anterior laryngeal polyp (Fig, D-E). Bronchial and tracheal biopsies revealed mild fibrosis, chronic inflammation, and a few neutrophils (consistent with nonspecific chronic bronchitis). Serology for ANA (Antinuclear Antibodies), ENA (Extraclable Nuclear Antigen), c-ANCA (cytoplasmic Anti-Neutrophil Cytoplasmic Antibodies), p-ANCA (perinuclear Anti-Neutrophilic Cytoplasmic Antibodies) , and sACE (serum Angiotensin-Converting Enzyme) was negative. Magnetic resonance imaging (MRI) confirmed diffuse tracheobronchial wall thickening. Spirometry indicated an obstructive pattern.

**Figure:**
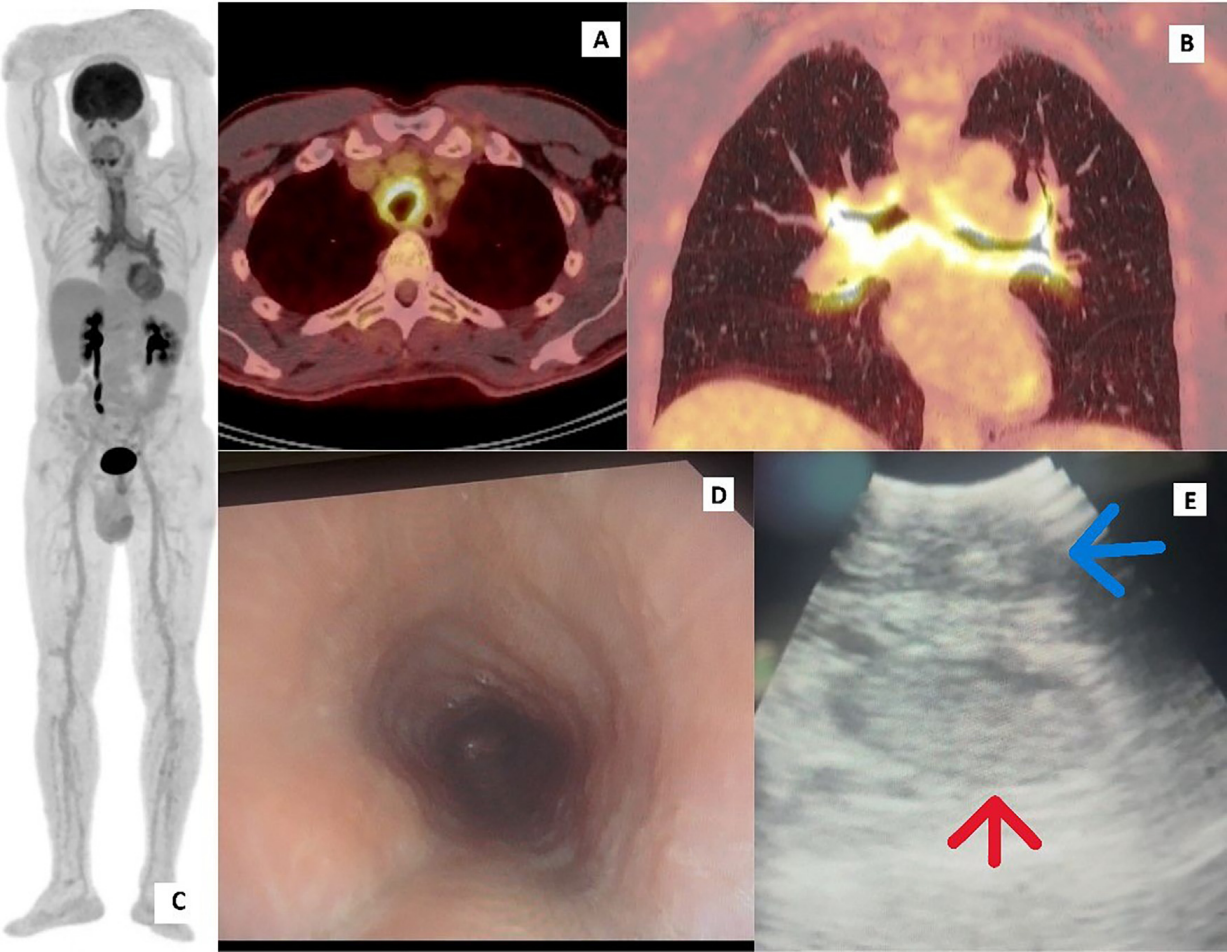
A, PET-CT scan demonstrating uptake of 18-FDG by the tracheal wall, sparing the posterior tracheal circumference (transverse view). B, 18-FDG uptake of the main and proximal bronchi symmetrically (coronal view). C, A full-body PET-CT scan showing the diffuse uptake of 18-FDG by the trachea and main bronchi. D, Bronchoscopic image of the trachea demonstrating diffuse mucosal oedema, sparing the posterior tracheal circumference. E, An EBUS image showcasing the thickened tracheal cartilage (blue arrow) and an enlarged paratracheal lymph node (red arrow). EBUS: Endobronchial Ultrasound 18-FDG: fluorine-18 fluorodeoxyglucose.

## ANSWER

These findings supported a diagnosis of relapsing polychondritis (RP) with isolated tracheobronchial involvement. Treatment with methylprednisolone (0.5 mg/kg daily) led to a rapid resolution of cough and fever. Infliximab (5 mg/kg every four weeks) was added as a steroid-sparing agent. One month after initiating treatment, the patient was asymptomatic.

Tracheobronchial involvement occurs in 30% to 50% of RP cases and can lead to life-threatening issues like airway stenosis and tracheobronchomalacia [1,2]. Isolated tracheobronchial involvement without auricular disease is rare and has been mainly reported in respiratory and radiology journals, often posing significant diagnostic challenges due to the absence of typical external cartilage manifestations [[3], [4], [5]]. PET-CT and MRI are valuable for assessing disease extent and treatment response. Early detection and immunosuppression are crucial in preventing irreversible airway damage.

## Competing interests

All authors declare they have no competing interests.
